# *TERT* promoter mutations and long telomere length predict poor survival and radiotherapy resistance in gliomas

**DOI:** 10.18632/oncotarget.6007

**Published:** 2015-11-09

**Authors:** Ke Gao, Gang Li, Yiping Qu, Maode Wang, Bo Cui, Meiju Ji, Bingyin Shi, Peng Hou

**Affiliations:** ^1^ Department of Endocrinology, The First Affiliated Hospital of Xi'an Jiaotong University, Xi'an 710061, the People's Republic of China; ^2^ Department of Neurosurgery, The First Affiliated Hospital of Xi'an Jiaotong University, Xi'an 710061, the People's Republic of China; ^3^ Department of Neurosurgery, Tangdu Hospital, Fourth Military Medical University, Xi'an 710038, the People's Republic of China; ^4^ Center for Translational Medicine, The First Affiliated Hospital of Xi'an Jiaotong University, Xi'an 710061, the People's Republic of China

**Keywords:** glioma, TERT promoter mutations, relative telomere length, poor survival, radiotherapy resistance

## Abstract

Increasing evidences have implicated somatic gain-of-function mutations at the telomerase reverse transcriptase (*TERT*) promoter as one of the major mechanisms that promote transcriptional activation of *TERT* and subsequently maintain telomere length in human cancers including glioma. To investigate the prognostic value of these mutations and telomere length, individually and their coexistence, in gliomas, we analyzed two somatic mutations C228T and C250T in the *TERT* promoter, relative telomere length (RTL), *IDH1* mutation and *MGMT* methylation in 389 glioma patients, and explored their associations with patient characteristics and clinical outcomes. Our data showed that C228T and C250T mutations were found in 17.0% (66 of 389) and 11.8% (46 of 389) of gliomas, respectively, and these two mutations were mutually exclusive in this cancer. Moreover, they were significantly associated with WHO grade. We also found that the RTL was significant longer in gliomas than in meningiomas and normal brain tissues (Median, 0.89 *vs*. 0.44 and 0.50; *P* < 0.001), and demonstrated that the RTL was strongly correlated with tumor recurrence. Importantly, *TERT* promoter mutations or long RTL caused a significantly poorer survival than *TERT* wild-type or short RTL. Coexisting *TERT* promoter mutations and long RTL were more commonly associated with poor patient survival than they were individually. Notably, the patients with *TERT* promoter mutations particularly C228T or long RTL were resistant to radiotherapy. Collectively, *TERT* promoter mutations and long RTL are not only prognostic factors for poor clinical outcomes, but also the predictors of radiotherapy resistance in gliomas.

## INTRODUCTION

Glioma is the most common primary brain tumor in adults, and patients with malignant glioma always have a poor prognosis due to the progressive overgrowth and diffuse invasion [1]. Although recent advances in standard therapy including surgical resection followed by radiation and/or chemotherapy, the prognosis is still disappointing [2]. Thus, a better understanding of the mechanisms underlying glioma pathogenesis may lead to more precise prognostic prediction and more effective therapies for this disease.

Telomerase, a ribonucleoprotein that consists of an RNA subunit and a telomerase reverse transcriptase (TERT) catalytic subunit, maintains telomere length through adding telomeric repeats to chromosome ends [3]. Telomeres are shortened with each cell division in normal cells, whereas they are continuously elongated by telomerase in cancer cells [4]. Increased telomerase activity is perceived to be one of the hallmarks of human cancers including gliomas, overcoming replicative telomere shortening and conferring unrestricted growth of cancer cells [4, 5]. The *TERT* promoter has been well demonstrated to be the most important element of telomerase expression/activity through transcriptional regulation [6]. Recently, recurrent mutations at two hotspots termed C228T and C250T in the *TERT* promoter have been identified in diverse cancers including gliomas [7–12]. These mutations create new binding motifs for Ets/TCF transcription factors and cause two- to four-fold increase in transcriptional activity [7], and have been regarded as one of the major mechanisms of telomerase activation in gliomas [12]. In addition, a very recent study reveals that the transcription factor GABP selectively binds and activates the mutant *TERT* promoter, also contributing to aberrant expression of *TERT* gene in multiple cancers, including gliomas [13].

In this study, we sought to investigate *TERT* promoter mutations and relative telomere length (RTL) in a large cohort of Chinese patients with well-characterized gliomas, and explore their associations with clinical outcomes of these patients.

## RESULTS

### Patient characteristics

A total of 389 glioma specimens were analyzed in this study, including 247 diffuse astrocytomas (DA), 44 oligodendrogliomas (OL), 46 oligoastrocytomas (OA) and 52 glioblastomas (GBM). The patient group was composed of 216 men and 173 women with a mean age of 44.9 ± 14.1 years (range: 18–81). Median overall survival time for the total was 40.3 months. Malignant glioma patients were managed according to ESMO clinical recommendations as described previously [14]. Briefly, the patients were divided into four groups: i) subsequent to surgery, 94 patients received adjuvant radiotherapy alone (35–50 Gy of craniospinal radiotherapy); ii) 33 patients received adjuvant chemotherapy alone (temozolomide, 150 mg/m2 for 5 days for first cycle, 200 mg/m2 for 5 days every 28 days, ≥6 cycles); iii) 104 patients received the combined therapy; iv) 99 patients did not receive further therapy. Analysis of *IDH1* mutations was performed in 330 tumor samples. One hundred and twenty patients (36.4%) harbored *IDH1* mutations, with 118 displaying R132H (CGT → CAT) mutation and 2 displaying R132C (CGT → TGT) mutation (Supplementary Figure S1A). For *MGMT* promoter methylation analysis, DNA from 330 tumor samples was investigated by MSP assay. Methylated *MGMT* promoter sequences were detected in 190 cases (57.6%) (Supplementary Figure S1B). Other patient characteristics were summarized in Table 1.

**Table 1:** Association of *TERT* promoter mutations and the RTL with clinicopathological characteristics in gliomas (*n* = 389)

Characteristics	*TERT* promoter mutations (*n* = 389)									RTL (*n* = 389)		
	Overall			C228T			C250T					
	Yes (%)	No (%)	*P*	Yes (%)	No (%)	*P*	Yes (%)	No (%)	*P*	≤0.62 (%)	>0.62 (%)	*P*
No. of Patients	112 (28.8)	277 (71.2)		66 (17.0)	323 (83.0)		46 (11.8)	343 (88.2)		143 (36.8)	246 (63.2)	
Gender												
Male	51 (23.6)	165 (76.4)	0.013	34 (15.7)	182 (84.3)	0.499	17 (7.9)	199 (92.1)	0.007	84 (38.9)	132 (61.1)	0.343
Female	61(35.3)	112(64.7)		32 (18.5)	141 (81.5)		29 (16.8)	144 (83.2)		59 (34.1)	114 (65.9)	
Age, years												
Mean	48	44	0.005	47	44	0.112	49	44	0.037	45	45	0.833
SD	13.7	14.1		14.4	14.1		12.8	14.2		13.1	14.8	
WHO grade												
I	5 (15.2)	28 (84.8)	0.039	1 (3.0)	32 (97.0)	0.072	4 (12.1)	29 (87.9)	0.858	11 (33.3)	22 (66.7)	0.685
II	40 (24.7)	122 (75.3)		22 (13.6)	140 (86.4)		18 (11.1)	144 (88.9)		65 (40.1)	97 (59.9)	
III	41 (32.3)	86 (67.7)		27 (21.3)	100 (78.7)		14 (11.0)	113 (89.0)		45 (35.4)	82 (64.6)	
IV	26 (38.8)	41 (61.2)		16 (23.9)	51 (76.1)		10 (14.9)	57 (85.1)		22 (32.8)	45 (67.2)	
Localization												
Frontal lobe	58 (33.9)	113 (66.1)	0.069	36 (21.1)	135 (78.9)	0.020	22 (12.9)	149 (87.1)	0.010	59 (34.5)	112 (65.5)	0.083
Temporal lobe	41 (29.1)	100 (70.9)		22 (15.6)	119 (84.4)		19 (13.5)	122 (86.5)		50 (35.5)	91 (64.5)	
Parietal lobe	19 (28.4)	48 (71.6)		12 (17.9)	55 (82.1)		7 (10.4)	60 (89.6)		22 (32.8)	45 (67.2)	
Occipital lobe	9 (39.1)	14 (60.9)		7 (30.4)	16 (69.6)		2 (8.7)	21 (91.3)		7 (30.4)	16 (69.6)	
Cerebellum	2 (11.8)	15 (88.2)		2 (11.8)	15 (88.2)		0 (0.0)	17 (100.0)		12 (70.6)	5 (29.4)	
Others	6 (14.3)	36 (85.7)		3 (7.1)	39 (92.8)		3 (7.1)	39 (92.8)		16 (38.1)	26 (61.9)	
Pathological diagnosis												
DA	66 (26.7)	181 (73.3)	0.157	37 (15.0)	210 (85.0)	0.006	29 (11.7)	218 (88.3)	0.767	91 (36.8)	156 (63.2)	0.496
OL	10 (22.7)	34 (77.3)		3 (6.8)	41 (93.2)		7 (15.9)	37 (84.1)		14 (31.8)	30 (68.2)	
OA	19 (41.3)	27 (58.7)		15 (32.6)	31 (67.4)		4 (8.7)	42 (91.3)		21 (45.6)	25 (54.3)	
GBM	17 (32.7)	35 (67.3)		11 (21.2)	41 (78.8)		6 (11.5)	46 (88.5)		17 (32.7)	35 (67.3)	
Recurrence^▼^												
Yes	61 (30.7)	138 (69.3)	0.171	34 (17.1)	165 (82.9)	0.225	27 (13.6)	172 (86.4)	0.498	57 (28.6)	142 (71.4)	0.005
No	31 (23.7)	100 (76.3)		17 (13.0)	114 (87.0)		14 (10.7)	117 (89.3)		58 (44.3)	73 (55.7)	
KPS^▼^												
≥ 80	41 (23.3)	135 (76.7)	0.050	22 (14.3)	132 (85.7)	0.648	29 (18.8)	125 (81.2)	0.001	61 (39.6)	93 (60.4)	0.105
< 80	51 (33.1)	103 (66.9)		29 (16.5)	147 (83.5)		12 (6.8)	164 (93.1)		54 (30.7)	122 (69.3)	
Seizures^▼^												
Yes	42 (30.2)	97 (69.8)	0.457	20 (14.4)	119 (85.6)	0.758	22 (15.8)	117 (84.2)	0.129	44 (31.7)	95 (68.3)	0.349
No	50 (26.2)	141 (73.8)		31 (16.2)	160 (83.8)		19 (9.9)	172 (90.1)		71 (37.2)	120 (62.8)	
Radiotherapy^▼^												
Yes	54 (27.3)	144 (72.7)		32 (16.2)	166 (83.8)		22 (11.1)	176 (88.9)		64 (32.3)	143 (67.7)	
No	38 (28.8)	94 (71.2)		19 (14.4)	113 (85.6)		19 (14.4)	113 (85.6)		51 (38.6)	81 (61.4)	
Chemotherapy^▼^												
Yes	37 (27.0)	100 (73.0)		21 (15.3)	116 (84.7)		16 (11.7)	121 (88.3)		50 (36.5)	87 (63.5)	
No	55 (28.5)	138 (71.5)		30 (15.5)	163 (84.5)		25 (13.0)	168 (87.0)		65 (33.7)	128 (66.3)	
*IDH1* mutation^▼^												
Yes	34 (28.3)	86 (71.7)	0.899	21 (17.5)	99 (82.5)	0.527	13 (10.8)	107 (89.2)	0.604	37 (30.8)	83 (69.2)	0.280
No	58 (27.6)	152 (72.4)		30 (14.3)	180 (85.7)		28 (13.3)	182 (86.7)		78 (37.1)	132 (62.9)	
*MGMT* methylation^▼^												
Yes	57 (30.0)	133 (70.0)	0.324	35 (18.4)	155 (81.6)	0.091	22 (11.6)	168 (88.4)	0.615	66 (34.7)	124 (65.3)	1.000
No	35 (25.0)	105 (75.0)		16 (11.4)	124 (88.6)		19 (13.6)	121 (86.4)		49 (35.0)	91 (65.0)	

Abbreviations: Relative telomere length (RTL); Diffuse astrocytoma (DA); Oligodendroglioma (OL); Oligoastrocytoma (OA); Glioblastoma (GBM); Karnofsky performance status (KPS);

▼ Only 330 patients have complete survival information.

### *TERT* promoter mutations and the RTL in gliomas

**Figure 1:**
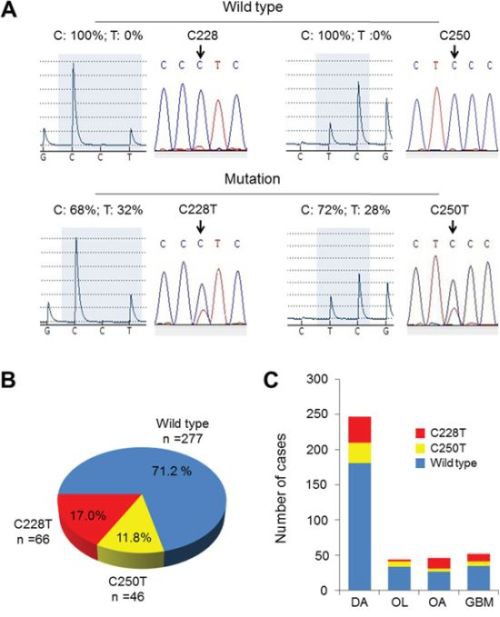
*TERT* promoter mutations in gliomas **A.** Shown are representative pyrograms (left) and electropherograms (right) of the *TERT* wild-type and two *TERT* promoter mutations as indicated for gliomas. **B.** Pie chart of C228T and C250T somatic mutation status in 389 primary gliomas. **C.** Frequency of *TERT* promoter mutations (C228T, red; C250T, yellow) in each tumor subtype. DA, diffuse astrocytoma; OL, oligodendroglioma; OA, oligoastrocytoma; GBM, glioblastoma.

Two hot-spot mutations (C228T and C250T) in the *TERT* promoter were investigated in a total of 389 glioma samples by Pyrosequencing and Sanger sequencing in this study. In Figure 1A, representative pyrograms and electropherograms of the two *TERT* promoter mutations in gliomas were shown. Figure 1B and 1C summarize *TERT* promoter mutations found in different subtypes of gliomas. These two mutations were collectively found in 28.8% (112 of 389) of gliomas. C228T and C250T mutations were found in 17.0% (66 of 389) and 11.8% (46 of 389) of gliomas, respectively. All mutations found in this study were heterozygous, and these two mutations were mutually exclusive in this cancer.

**Figure 2:**
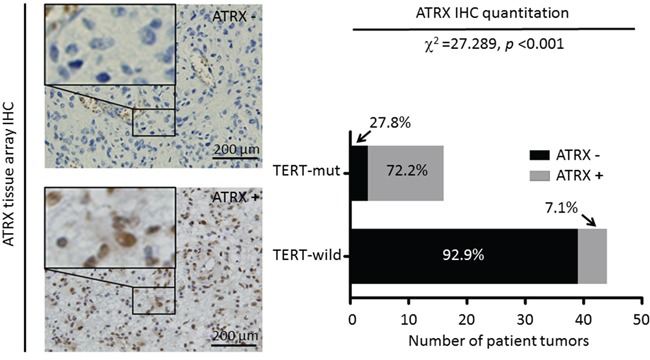
Differential expression of ATRX in gliomas Immunohistochemical (IHC) assays were performed to detect ATRX activation using anti-ATRX antibody. Shown are representative IHC images of glioma tissues with or without ATRX staining (left panel) (magnification, ×200). A comparison of the proportion of patients with *TERT* promoter mutations or *TERT* wild-type associated with positive immunostaining of ATRX (right panel). The relationship between them was evaluated using Fisher's exact test.

Comparing with previous reports [12], the prevalence of *TERT* promoter mutations is relatively lower in the present study. To support this finding, ATRX activation was detected by IHC assay in 60 glioma tissues, including 18 cases with *TERT* promoter mutations and 42 *TERT* wild-type cases. As shown in Figure 2, positive immunostaining of ATRX in cases with *TERT* promoter mutations and *TERT* wild-type was 72.2% (13/18) and 7.1% (3/42), respectively. There was a significantly positive relationship between *TERT* promoter mutations and ATRX activation (Χ^2^ = 27.289, *P* < 0.001) in gliomas, as supported by the previous studies that *TERT* promoter and *ATRX* mutations (usually caused ATRX inactivation) were mutually exclusive [9, 15].

Next, the RTL was measured in 389 gliomas, 50 meningiomas and 8 normal brain tissues by using real-time quantitative PCR. As shown in Supplementary Figure S2A, the RTL was significantly longer in gliomas than that in meningiomas and normal brain tissues (Median, 0.89 *vs*. 0.44 and 0.50; *P* < 0.001). In addition, we also investigated the relationship between *TERT* promoter mutations and the RTL in gliomas. The results showed that the RTL was significantly shorter in the cases with *TERT* promoter mutations than in the cases with *TERT* wild-type (*P* = 0.017) (Supplementary Figure S2B), indicating that other molecular mechanisms may be involved in regulating telomere length in the latter. It has been reported that many *TERT* wild-type gliomas harbor highly frequent ATRX mutations, which induce the Alternative Lengthening of Telomeres (ALT) phenotype [15, 16]. As expected, we found highly frequent ATRX inactivation (∼92.9%) in *TERT* wild-type gliomas in the present study (Figure 2), and demonstrated that the RTL was significantly longer in ATRX-negative tumors than that in ATRX-positive tumors (Median, 0.90 vs. 0.49; *P* = 0.002) (Supplementary Figure S2C). These findings suggest that telomere length may also be regulated by ALT phenotype such as ATRX inactivation in *TERT* wild-type tumors, as supported by the previous studies [16]. A recent study showed that gliomas with *TERT* promoter mutations had shorter telomere length compared to *TERT* wild-type gliomas [17], which was consistent with our findings. In addition, coexisting *TERT* promoter mutations and long RTL were also found in 55 of 330 (16.7%) gliomas.

### Association of *TERT* promoter mutations and variable RTL with clinicopathological characteristics of glioma patients

The relationship between *TERT* promoter mutations and patient characteristics was summarized in Table 1. The results showed that there was a significantly positive association of *TERT* promoter mutations with WHO grade (*P* = 0.039). The prevalence of *TERT* promoter mutations was only 15.2% (5 of 33) in patients with grade I gliomas, whereas these mutations were found in 24.7% (40 of 162), 32.3% (41 of 127) and 38.8% (26 of 67) in patients with grade II-IV gliomas, respectively. These results suggest that *TERT* promoter mutations were more frequent with high-grade gliomas. To investigate the relationship between the RTL with patient characteristics, we defined the upper limit of the overall 95% confidence interval (CI, 0.45–0.62) for 50 meningioma samples as cut-off value. Glioma patients were then categorized into two groups by use of this cut-off point: short telomere (≤0.62) and long telomere (>0.62). Also shown in Table 1, the RTL was only significantly associated with tumor recurrence (*P* < 0.001).

To assess the association of *TERT* promoter mutations and the RTL with gender, age, WHO grade, pathological diagnosis, recurrence, seizures, KPS score, *IDH1* mutation, and *MGMT* methylation, we conducted multiple multivariable logistic regressions. As shown in Table 2, *TERT* promoter mutations remained closely associated with WHO grade (OR = 1.81, 95% CI = 1.23–2.66; *P* = 0.003). The RTL was still significantly associated with tumor recurrence (OR = 1.70, 95% CI = 1.00–2.89; *P* = 0.049) after adjustment. In addition, the RTL was significantly associated with age (OR = 2.24, 95% CI = 1.09–4.60; *P* = 0.029).

**Table 2:** Multivariable analysis of characteristics of glioma patients divided according to *TERT* promoter mutations and the RTL (*n* = 330)

Characteristics	*TERT* promoter mutations						RTL	
	Overall		C228T		C250T			
	OR* (95% CI)	*P*	OR* (95% CI)	*P*	OR* (95% CI)	*P*	OR* (95% CI)	*P*
Gender	2.12 (1.26–3.56)	0.005	1.26 (0.67–2.36)	0.478	3.19 (1.51–6.75)	0.002	1.33 (0.83–2.14)	0.243
Age^1^	1.94 (1.00–3.76)	0.052	2.11 (0.96–4.64)	0.062	1.37 (0.55–3.42)	0.503	2.24 (1.09–4.60)	0.029
WHO grade^2^	1.81 (1.23–2.66)	0.003	1.52 (0.95–2.44)	0.083	1.80 (1.08–3.01)	0.025	0.95 (0.67–1.33)	0.751
Pathological diagnosis^3^	0.94 (0.72–1.22)	0.643	1.08 (0.79–1.47)	0.628	0.80 (0.55–1.17)	0.248	0.95 (0.74–1.22)	0.680
Recurrence	1.46 (0.79–2.70)	0.225	1.19 (0.56–2.54)	0.646	1.54 (0.67–3.57)	0.311	1.70 (1.00–2.89)	0.049
Seizures	1.52 (0.88–2.63)	0.133	0.99 (0.51–1.94)	0.980	2.13 (0.99–4.56)	0.052	1.23 (0.75–2.01)	0.417
KPS^4^	2.68 (1.50–4.80)	0.001	1.02 (0.51–2.04)	0.964	6.33 (2.73–14.71)	<0.001	1.32 (0.79–2.20)	0.289
*IDH1* mutation	0.96 (0.55–1.71)	0.900	1.50 (0.76–2.98)	0.246	0.53 (0.24–1.20)	0.131	1.45 (0.86–2.45)	0.168
*MGMT* methylation	1.27 (0.74–2.18)	0.391	1.67 (0.84–3.30)	0.142	0.81 (0.39–1.70)	0.577	0.99 (0.60–1.61)	0.951
RTL	0.58 (0.34–1.01)	0.053	0.43 (0.22–0.82)	0.011	1.07 (0.49–2.32)	0.871	—	—

* OR: odds ratio with 95% confidence interval;

1 Age (≥60; <60);

2 WHO grade (I; II; III; IV);

3 Pathological diagnosis (Diffuse astrocytoma; Oligodendroglioma; Oligoastrocytoma; Glioblastoma);

4 KPS (≥80; <80).

### Effect of *TERT* promoter mutations and variable RTL on poor patient survival

**Figure 3:**
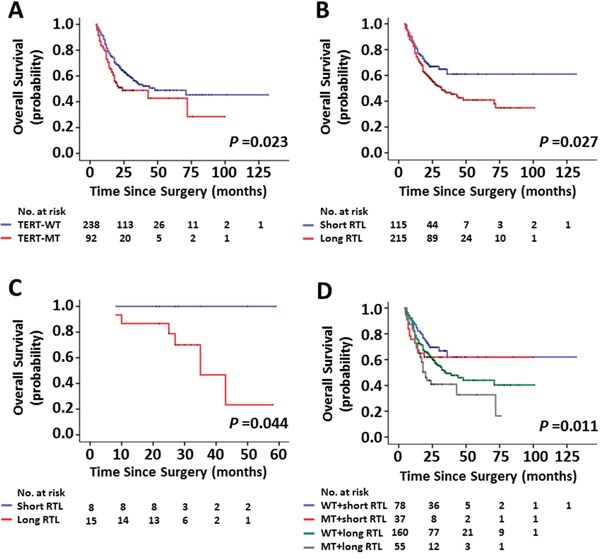
Kaplan-Meier analysis of overall survival for glioma patients assessed for the mutational status of *TERT* promoter and the RTL individually or in combination **A.** The patients with *TERT* promoter mutations had a worse survival than those without mutations. Long RTL caused a poorer overall survival than short RTL in all patients **B.** and in the patients with grade I gliomas **C.** respectively. **D.** Overall survival in glioma patients where patient groups were defined by the mutational status of *TERT* promoter and the RTL. The patients with coexisting *TERT* promoter mutations and long RTL had much worse survival than others. WT and MT represent the *TERT* promoter wild-type and -mutated tumors, respectively.

Whether *TERT* promoter mutations and variable RTL are indeed associated with poor overall survival (OS), as suggested by their association with clinicopathologic features of glioma patients, was subsequently investigated by univariate survival analysis. As shown in Table 3, *TERT* promoter mutations (HR = 1.49, 95% CI, 1.05–2.10; *P* = 0.026) and long RTL (HR = 1.50, 95% CI, 1.04–2.17; *P* = 0.030) were significantly associated with poor survival in glioma patients. Next, we used Kaplan-Meier survival analysis to further determine the effect of *TERT* promoter mutations and variable RTL on patient survival. As expected, the patients with *TERT* promoter mutation (*P* = 0.023) or long RTL (*P* = 0.027) had significantly poorer survival than those without mutations or with short RTL (Figure 3A and 3B). Accordingly, 1-, 2- and 3-year OS rates were worse in the former that in the latter (Table 4). Although the number was small, our data showed that the RTL significantly affected the survival in the patients with grade I gliomas. The patients with long RTL had worse survival than those with short RTL (*P* = 0.044) (Figure 3C). In addition, we demonstrated that the patients with coexisting *TERT* promoter mutations and long RTL had much worse survival than others (Table 4 and Figure 3D). Given that the majority of gliomas in the present study are astrocytomas, similar analysis has been done only in astrocytomas. Similar to the findings in Figure 3, *TERT* promoter mutations and longer RTL caused a significantly poorer survival of astrocytoma patients than *TERT* wild-type or short RTL (Supplementary Figures S3A and S3B). In addition, we also analyzed the relationship between *TERT* promoter status and patient survival for other histological types including oligodendroglioma, oligoastrocytoma and glioblastoma. Similar results were also found, except for oligoastrocytoma because of the limited number (Supplementary Figure S4).

**Table 3:** Prognostic value of *TERT* promoter mutations and the RTL in univariate and multivariate cox regression analysis in glimoas (*n* = 330)

Characteristics	Univariateanalysis		Multivariate analysis forTERT		Multivariate analysis for RTL	
	Hazard Ratio (95% CI)	*P*	Hazard Ratio (95% CI)	*P*	Hazard Ratio (95% CI)	*P*
Gender	1.15 (0.84–1.59)	0.385	1.19 (0.85–1.64)	0.310	1.17 (0.85–1.63)	0.338
Age	3.24 (2.26–4.64)	<0.001	1.04 (1.02–1.04)	<0.001	1.03 (1.02–1.04)	<0.001
Pathological diagnosis^1^	1.09 (0.94–1.27)	0.271	1.13 (0.97–1.32)	0.122	1.14 (0.98–1.33)	0.091
*IDH1* mutations	0.59 (0.41–0.84)	0.004	0.48 (0.40–0.89)	0.018	0.68 (0.47–0.99)	0.022
*MGMT* methylation	0.60 (0.44–0.83)	0.002	0.51 (0.36–0.71)	<0.001	0.51 (0.36–0.72)	<0.001
Radiotherapy	0.89 (0.64–1.23)	0.467	0.74 (0.53–1.04)	0.084	0.66 (0.46–0.94)	0.020
Chemotherapy	1.75 (1.27–2.42)	0.001	2.09 (1.49–2.94)	<0.001	2.23 (1.58–3.16)	<0.001
*TERT* promoter mutations	1.49 (1.05–2.10)	0.026	1.39 (1.13–2.17)	0.020	-	-
RTL	1.50 (1.04–2.17)	0.030	-	-	1.72 (1.18–2.50)	0.005

1 Pathological diagnosis (Diffuse astrocytoma; Oligodendroglioma; Oligoastrocytoma; Glioblastoma)

**Table 4:** Overall survival by grouping with *TERT* promoter mutations and the RTL (*n* = 330)

Characteristics	Overall survival rate (%)			Overall survival time (month)	
	1 year (95% CI)	2 years (95% CI)	3 years (95% CI)	Median	95% CI
*TERT* promoter mutations					
No (WT)	81.5 (76.6–86.4)	64.3 (58.0–70.6)	53.3 (45.9–60.7)	48.2	15.6–68.9
Yes (MT)	72.8 (63.8–81.8)	48.8 (38.0–59.6)	42.7 (28.0–57.4)	23.5	3.7–44.3
RTL					
Short (≤0.62)	82.6 (75.7–89.5)	66.9 (58.1–75.7)	61.0 (49.6–72.4)	Not reached	—
Long (>0.62)	77.2 (71.5–82.9)	56.6 (49.7–63.5)	46.7 (39.1–54.3)	32.0	20.2–43.8
Combinations					
WT and Short RTL	87.2 (79.8–94.6)	69.6 (59.2–80.0)	62.0 (48.1–75.9)	Not reached	—
MT and Short RTL	73.0 (58.7–87.3)	61.9 (46.2–77.6)	61.9 (46.2–77.6)	Not reached	—
WT and Long RTL	78.8 (72.5–85.1)	61.7 (54.1–69.3)	49.3 (40.5–58.1)	35.0	20.2–49.8
MT and Long RTL	72.7 (60.9–84.5)	40.9 (27.2–54.6)	32.7 (14.7–50.7)	20.0	16.2–23.8

Abbreviations: WT, *TERT* promoter wild type; MT, *TERT* promoter mutations; RTL, relative telomere length

Next, we attempted to explore whether the prognostic value of *TERT* promoter mutations and variable RTL as found in univariate analysis is attributable to their association with other factors or whether they contribute independently to survival. Thus, Cox multivariate regression analysis was performed in this study. Also shown in Table 3, *TERT* promoter mutations (HR = 1.39; 95% CI, 1.13–2.17; *P* = 0.020) and long RTL (HR = 1.72; 95% CI, 1.18–2.50; *P* = 0.005) are predictors of poor OS for glioma patients as independently variable with respect to gender, age, pathological diagnosis, *IDH1* mutations, *MGMT* methylation, radiotherapy and chemotherapy.

Notably, we also demonstrated that the patients with symptoms of seizure had better survival than patients without the symptoms (*P* = 0.012) (Supplementary Figure S5). There is a strong possibility that the majority of the patients with symptoms of seizure are diagnosed as low-grade tumors in this study, accounting for 59.7% of all cases with symptoms of seizure, as supported by a previous study [18].

### Impact of *TERT* promoter mutations and the RTL on outcomes of radiotherapy and chemotherapy in glioma patients

**Figure 4:**
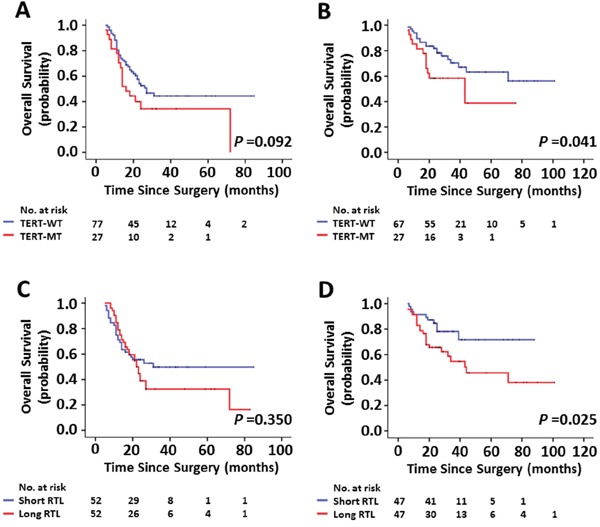
Kaplan-Meier estimates of overall survival by the mutational status of *TERT* promoter and the RTL **A, C.** Represent Kaplan-Meier analysis of 104 patients receiving both radiotherapy and chemotherapy after surgery. **B, D.** Represent Kaplan-Meier analysis of 94 patients only receiving postoperative radiotherapy.

**Figure 5:**
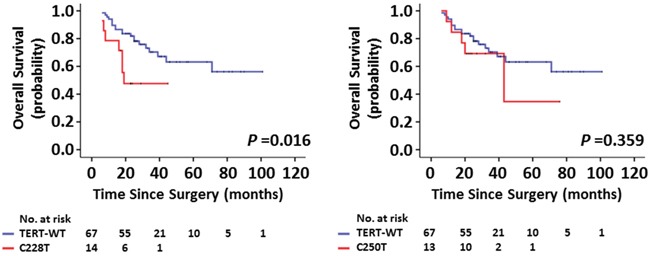
Effect of C228T and C250T mutations individually on radiotherapy response Represent Kaplan-Meier analysis of 94 patients only receiving postoperative radiotherapy grouped by C228T (left) and C250T (right) mutations, respectively.

As adjuvant therapy after surgical resection for glioma, radiotherapy and chemotherapy can significantly improve the patient OS [19]. In this study, we tested the effect of *TERT* promoter mutations and the RTL on outcomes of radiotherapy and chemotherapy in a cohort of gliomas. As shown in Figure 4A, although the difference did not reach statistical significance, *TERT* promoter mutations were associated with lower 2-year OS rate in the patients receiving both radiotherapy and chemotherapy compared with *TERT* wild-type (34.3% ± 9.8% *vs*. 52.6% ± 5.9%, *P* = 0.092). Further analysis revealed that these mutations significantly affected 2-year OS rate in the patients only receiving radiotherapy (58.3% ± 9.6% *vs*. 81.8% ± 4.8%, *P* = 0.041) (Figure 4B), whereas they did not affect 2-year OS rate in the cases receiving chemotherapy compared with *TERT* wild-type (*P* = 0.304, figure not shown). Next, to divide the patients into two groups, we chose median RTL for all cases receiving radiotherapy and/or chemotherapy as cut-off values. As shown in Figure 4C, long RTL did not significantly affected 2-year OS rate in the patients receiving both radiotherapy and chemotherapy than short RTL (*P* = 0.350). Similar to *TERT* promoter mutations, long RTL caused a significantly poorer radiotherapy outcome (2-year OS rate: 65.6% ± 7.0% *vs*. 84.7% ± 5.4%, *P* = 0.025) in the patients only receiving radiotherapy than short RTL (Figure 4D). Moreover, we similarly did not find significant effect of long RTL on chemotherapy outcome in these patients (*P* = 0.824, figure not shown). Similarly, the astrocytoma patients with *TERT* promoter mutations or long RTL were also resistant to radiotherapy (Supplementary Figures S3C and S3D). To further clarify the role of *TERT* promoter mutations in radiotherapy outcome, C250T and C228T mutations were separately analyzed for their correlation with patients' response to radiotherapy. The results showed that C228T mutation, but not C250T mutation, significantly affected patients' response to radiotherapy (Figure 5).

**Figure 6:**
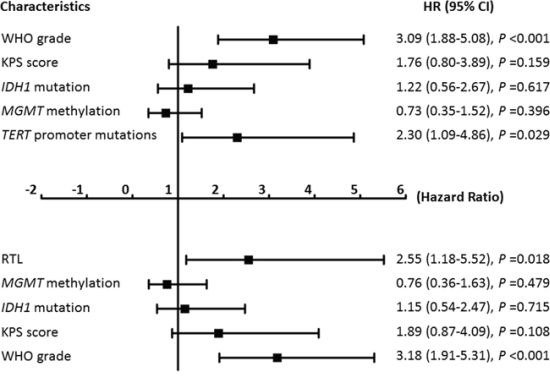
Multivariate Cox regression analysis Multivariate Cox regression analysis of tumor characteristics *TERT* promoter mutations and the RTL on overall survival in the patients (*n* = 94) only receiving postoperative radiotherapy.

Increasing evidences have demonstrated that *IDH1* mutations and *MGMT* methylation are associated with survival benefit in gliomas [20, 21]. This is as supported by our data that the cases with *IDH1*-mutated or *MGMT*-methylated tumors had better survival than those with *IDH1*-wild type or *MGMT*-unmethylated tumors (Supplementary Figure S6). Thus, we performed a Cox multivariate regression analysis to determine whether these two genetic events contribute independently to radiotherapy outcome of glioma patients. As shown in Figure 6, *TERT* promoter mutations (HR = 2.30; 95% CI, 1.09–4.86; *P* = 0.029) and long RTL (HR = 2.55; 95% CI, 1.18–5.52; *P* = 0.018) were two predictors of poor survival in glioma patients receiving radiotherapy as an independently variable with respect to WHO grade, KPS score, *IDH1* mutations and *MGMT* methylation, respectively. Collectively, our data suggest that glioma patients with *TERT* promoter mutation and long RTL are resistant to postoperative radiotherapy.

## DISCUSSION

In this study, we found 28.8% (112 of 389) glioma patients harbored *TERT* promoter mutations, which was relatively lower than previously reported [12]. However, we found highly frequent ATRX inactivation (∼92.9%) in *TERT* wild-type gliomas, and there was a significantly inverse relationship between *TERT* promoter mutations and ATRX inactivation. These observations suggest that each of these two molecular events plays a relatively independent role in maintaining the immortalization of glioma cells, ultimately contributing to glioma tumorigenesis. Given that *TERT* promoter mutations have been widely found in diverse cancers [7–12], these mutations are likely to be one of the mechanisms of telomerase activation in this cancer. As described in other cancers such as melanomas, thyroid cancer, bladder cancer and laryngeal cancer [7, 22–24], the present study and previous study [12] confirmed that C228T and C250T mutations were mutually exclusive in gliomas, suggesting that each of them is individually sufficient to play a significant oncogenic role in glioma pathogenesis. Importantly, these mutations have been demonstrated to be absent in benign tumors and normal subjects [7, 8, 24], implicating that they may be potentially valuable diagnostic and prognostic markers in human cancers including gliomas.

Considering the association of *TERT* promoter mutations with poor survival of primary gliomas [25–27], we investigated the effect of *TERT* promoter mutations on the prognosis of a cohort of 330 glioma patients in this study. As expected, *TERT* promoter mutations were closely associated with WHO grade, suggesting that these mutations may contribute to clinical outcomes of glioma patients. It is more noteworthy that these mutations predict poor patient survival, which was consistent with a previous study [11, 25–27]. These observations suggest that *TERT* promoter mutations play a critical role in glioma pathogenesis and are believed to be linked with the progression of this cancer.

As “mitotic clock”, telomere length reflects the life of differentiated somatic cells [28]. Over the years, telomere length measurement has been widely used as a marker for cell proliferation [3, 29]. Although the current findings are still in debate, telomere length variation is strongly implicated in the process of tumorigenesis [30]. In recent years, although there is still controversy, variable telomere length in peripheral blood leukocytes has been demonstrated to be associated with an increased risk of various cancers [31, 32], including gliomas [33]. However, only one study has addressed the link of telomere length and histopathologic parameters and patient survival in a limited number of glioblastomas, revealing that telomere length is associated with age and KPS score, as well as weakly contributes to patient survival [34]. In the present study, we measured telomere length in a cohort of 330 gliomas and investigated its association with clinopathologic characteristics and prognosis of glioma patients. Our data showed that telomere length was significantly correlated with age and tumor recurrence. Importantly, long telomere length is an independent factor for predicting poor patient survival. It is well known that patients with lower-grade gliomas always have longer survival times than patients with high-grade gliomas. However, in fact, a small number of patients with lower-grade gliomas have also very poor survival. In this study, we measured the RTL in 23 grade I tumors, which are considered to be “borderline tumor” and majority of scholars believe that it can be cured after surgery [1], and investigated the association of the RTL with patient survival. Our data demonstrated that the RTL significantly affected patient survival. None of patients with short RTL were dead, whereas 6 of 15 (40%) patients with long RTL were dead. These results suggest that telomere length may be an important predictor for clinical outcomes of the patients with low-grade gliomas.

Meanwhile, our data also showed that coexisting *TERT* promoter mutations and long RTL were significantly associated with poor patient survival than they were individually. Importantly, we observed that *TERT* promoter mutations particularly C228T and long telomere length significantly impacted radiotherapy outcome in glioma patients, which appears independent of WHO grade, KPS score, *IDH1* mutations and *MGMT* methylation. Although the exact mechanisms remain unclear, we have sound reasons to believe that these two genetic alterations can be used as useful biomarkers for predicting outcome of postoperative radiotherapy in gliomas.

In summary, we investigated *TERT* promoter mutations and alteration of telomere length in a large cohort of primary gliomas in Northwest China, and demonstrated that these mutations and long telomere length were closely associated with aggressive tumor behaviors and poor patient survival. Moreover, to our knowledge, the present study is the first to show that *TERT* promoter mutations and long telomere length affect radiotherapy outcome in glioma patients. Collectively, these observations raise the possibility that these two molecular events may be one of major driving forces for the progression of glioma, and may be potentially valuable prognostic markers for glioma patients.

## MATERIALS AND METHODS

### Patients and tissue samples

Gliomas (*n* = 389), benign meningiomas (*n* = 50) and normal brain tissues (*n* = 8) (form cerebral contusion and laceration patients) were randomly obtained at the First Affiliated Hospital of Xi'an Jiaotong University and the Tangdu Hospital of The Fourth Military Medical University between January 2004 and September 2013. Histopathological diagnosis was confirmed by at least two senior neuropathologists according to the World Health Organization (WHO) classification [1]. This study was approved by the local ethics committee, and written informed consent was obtained from all patients.

### DNA extraction

All tissues sections were reviewed by board-certified pathologists to ensure that ≥50% of the cells used for DNA purification were neoplastic. Genomic DNA was extracted from *fresh* or frozen *tissues by* using standard procedures of protease K digestion, phenol/chloroform extraction and ethanol precipitation. Genomic DNA was extracted from paraffin-embedded tissues as previously described [35]. Briefly, after a treatment for 8 to 10 h at room temperature with xylene to remove paraffin, tissues were digested with 1% sodium dodecyl sulfate (SDS) and 0.5 mg/ml proteinase K at 48°C for 48 h, with addition of several spiking aliquots of concentrated proteinase K to facilitate digestion. DNA was then isolated after standard phenol/chloroform extraction and ethanol precipitation protocols.

### Detection of *TERT* promoter and *IDH1* mutations

Two hot-spot somatic mutations (C228T and C250T) in the *TERT* core promoter were examined by pyrosequencing assay using the primers and conditions previously described [36]. Sanger sequencing was subsequently used to confirm the results of pyrosequencing using the same primer pair as for pyrosequencing assay. A fragment of 129 bp length spanning the catalytic domain of *IDH1* including codon 132 was amplified by PCR with the following primers: 5′-CGG TCT TCA GAG AAG CCA TT-3′ (forward) and 5′-GCA AAA TCA CAT TAT TGC CAA C-3′ (reverse) as previously described [37]. PCR products were analyzed by Sanger sequencing.

### Relative telomere length (RTL) measurement

Relative telomere length (RTL) was determined by real-time quantitative PCR as previously described [38]. Theoretically, the RTL can be measured using the primers that hybridize the telomeric hexamer repeats, because the number of binding sites for the primers increases as average telomere length increases. Two telomere primer sequences were as follows: tel-1,5′-CGG TTT GTT TGG GTT TGG GTT TGG GTT TGG GTT TGG GTT-3′, and tel-2,5′-GGC TTG CCT TAC CCT TAC CCT TAC CCT TAC CCT TAC CCT-3′. A single copy gene, *36B4*, was used as a reference. The primer sequences were 5′-CAG CAA GTG GGA AGG TGT AAT CC-3′ (36B4u) and 5′-CCC ATT CTA TCA TCA ACG GGT ACA A-3′ (36B4d). The reactions were carried out in a total of volume of 20 μl containing 25 ng of genome DNA, 1 × Maxima SYBR Green (SYBR Premix Ex Taq^TM^ II, TAKARA), 270 nM tel-1 and 900 nM tel-2 for telomere or 300 nM 36B4u and 500 nM 36B4d for reference gene *36B4*. The standard curve was established using serial dilutions of normal leukocyte DNA with a quantity range of 3.12 to 50 ng. Amplification conditions and RTL measure were described previously [38].

### Analysis of *MGMT* methylation

Genomic DNA was subjected to bisulfite treatment as described previously [35]. Next, Methylation-Specific PCR (MSP) was performed to evaluate methylation status of *MGMT* promoter with primers specific for either methylated or unmethylated DNA, as previously described [19]. The primer sequences for the methylated reaction were 5′-TTT CGA CGT TCG TAG GTT TTC GC-3′ (forward) and 5′-GCA CTC TTC CGA AAA CGA AAC G-3′ (reverse), and for the unmethylated reaction they were 5′-TTT GTG TTT TGA TGT TTG TAG GTT TTT GT-3′ (forward) and 5′-AAC TCC ACA CTC TTC CAA AAA CAA AAC A-3′ (reverse). The annealing temperature was 59°C. Normal leukocyte DNA treated *in vitro* with *Sss*I methyltransferase (New England Biolabs, Beverly, MA) was used as a positive control for methylated alleles of *MGMT*, and untreated normal leukocyte DNA was used as a negative control. The PCR products were electrophoresed on a 1.2% agarose gel and visualized under UV illumination using an ethidium bromide stain.

### Immunohistochemical (IHC) analysis of ATRX

IHC assays were performed as previously described [39]. In brief, paraffin-embedded sections (5 μm) were deparaffinized and rehydrated in a graded series of ethanol, and washed in distilled water. After antigen retrieval and blocking, the sections were incubated with anti-ATRX antibody (Abcam, ab97508; 1:300 dilution) overnight at 4°C. Slides were then counterstained with hematoxylin. The mutational status of *TERT* promoter was scored in a double-blinded manner.

### Statistical analysis

The Mann-Whitney *U* test was used to compare the RTL between glioma tissues and control subjects. The correlation between *TERT* promoter mutations and the RTL with clinicpathological characteristics was analyzed by Fisher's exact probability test (two-sided) or Chi-square test. Multivariate models were developed that adjusted for the most important covariates, including gender, age, WHO grade, pathological, disease recurrence, seizures, KPS score, *IDH1* mutation and *MGMT* methylation. Survival length was determined from the day of primary tumor surgery to the day of death or last clinical follow-up. Kaplan–Meier method was used for survival analysis grouping with *TERT* promoter mutations or appropriate cut-off value of RTL. Differences between curves were analyzed using the log-rank test. Multivariate Cox regression analysis was used to evaluate the effect of *TERT* promoter mutations and the RTL on survival and resistance to radiotherapy. All statistical analyses were performed using the SPSS statistical package (11.5, Chicago, IL, USA). *P* values < 0.05 were considered significant.

## SUPPLEMENTARY FIGURES


