# Supplementary material for: TERT promoter mutations and long telomere length predict poor survival and radiotherapy resistance in gliomas
Source: Oncotarget. 2015 Nov 9;7(8):8712–25. doi: 10.18632/oncotarget.6007 (PMC4890999; doi:10.18632/oncotarget.6007)
Supplement: Supplementary file 1 [file oncotarget-07-8712-s001.pdf]

SUPPLEMENTARY FIGURES

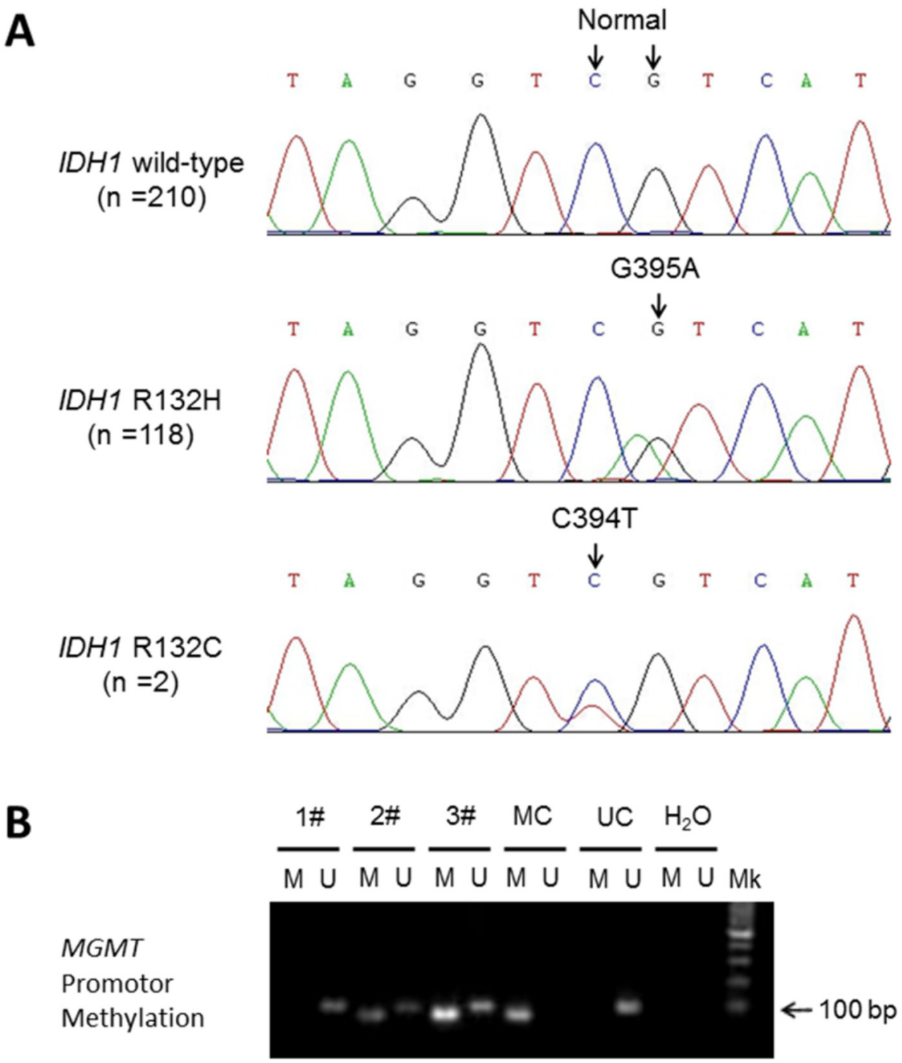

Supplementary Figure S1: *IDH1* mutations and MGMT promoter methylation in gliomas. A. Shown are representative electropherograms of the *IDH1* wild-type and –mutated tumors. **B.** Methylation status of the *MGMT* promoter in gliomas was determined by a MSP assay. Normal leukocyte DNA treated *in vitro* with *Sss*I methyltransferase was used as a positive control for methylation (MC), untreated normal leukocyte DNA was used as a negative control for methylation (UC), and water as a negative PCR control. M and U represent the presence of methylated and unmethylated genes, respectively. Mk represents the molecular-weight marker.

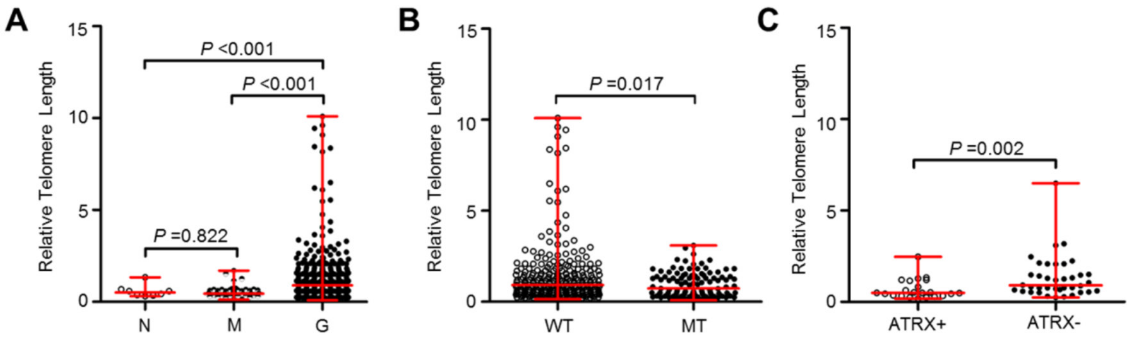

Supplementary Figure S2: Measurement of relative telomere length (RTL) in gliomas. A. The RTL corresponding to each individual case of normal brain tissues (N), meningiomas (M) and gliomas (G). **B.** The relationship between the RTL and *TERT* promoter mutations in gliomas. WT and MT represent the *TERT* wild-type and –mutated tumors, respectively. **C.** The association of the RTL with the status of ATRX in 60 gliomas. Horizontal lines represent the median and the minimum to maximum value. ATRX+ and ATRX- represent the ATRX-positive and -negative tumors, respectively.

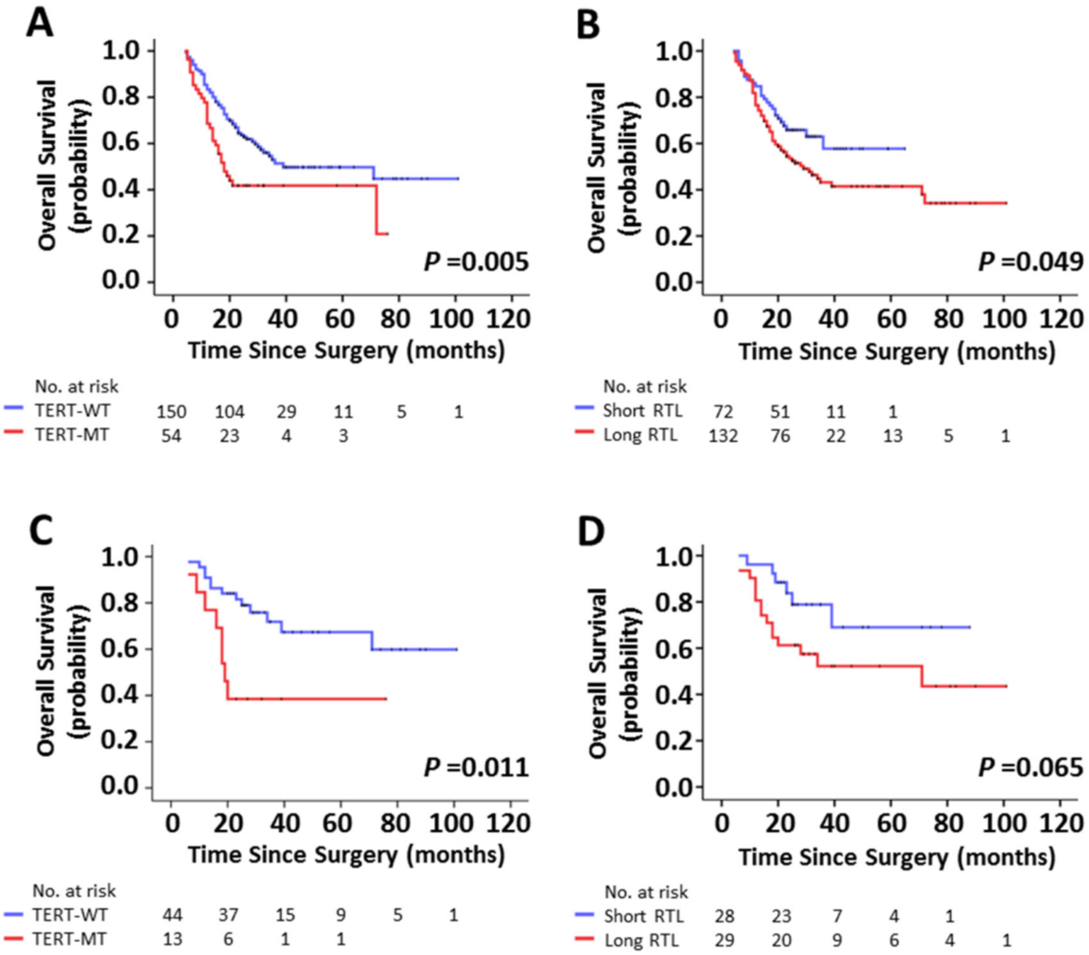

Supplementary Figure S3: Kaplan-Meier analysis of overall survival for astrocytoma patients assessed for the mutational status of *TERT* promoter and the RTL individually. A, B. The 204 astrocytoma patients with *TERT* promoter mutations and longer telomere had worse survival. **C, D.** Represent Kaplan-Meier analysis of 57 astrocytoma patients only receiving postoperative radiotherapy. WT and MT represent the *TERT* wild-type and -mutated tumors, respectively.

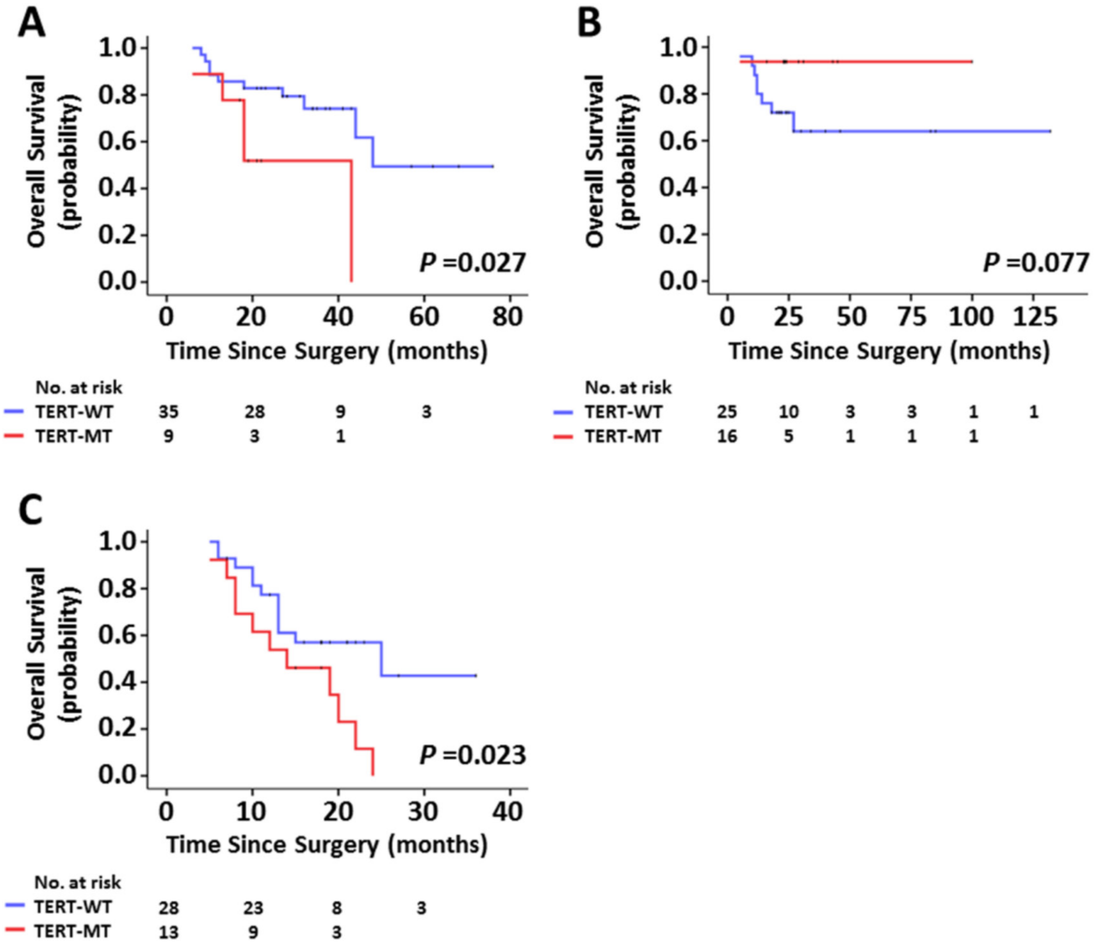

Supplementary Figure S4: Kaplan-Meier analysis of overall survival for the patients with oligodendroglioma A. oligoastrocytoma B. and glioblastoma C. by mutational status of *TERT* promoter.

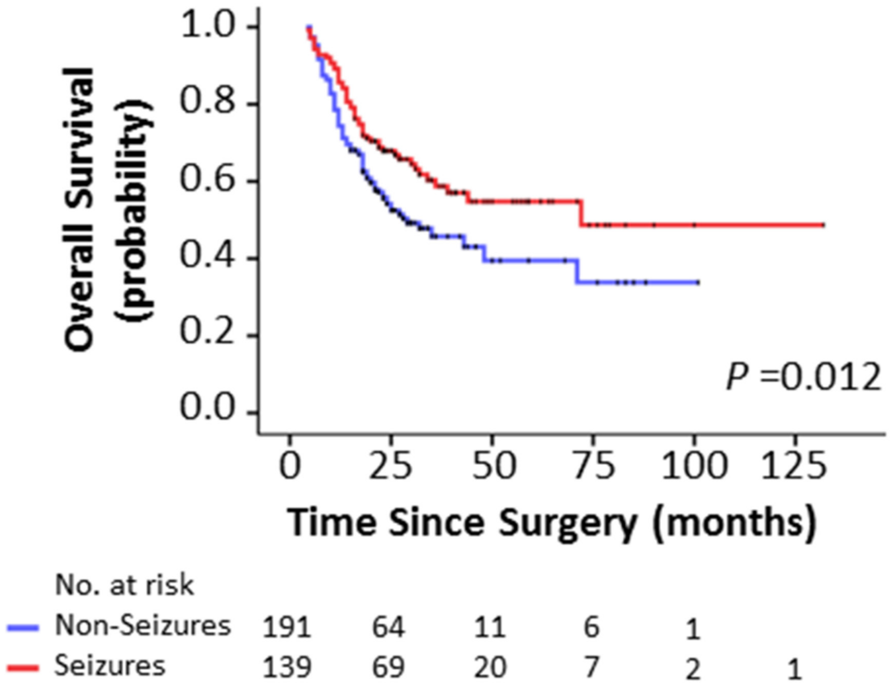

Supplementary Figure S5: Kaplan-Meier estimates of overall survival for 330 glioma patients by symptoms of seizure.

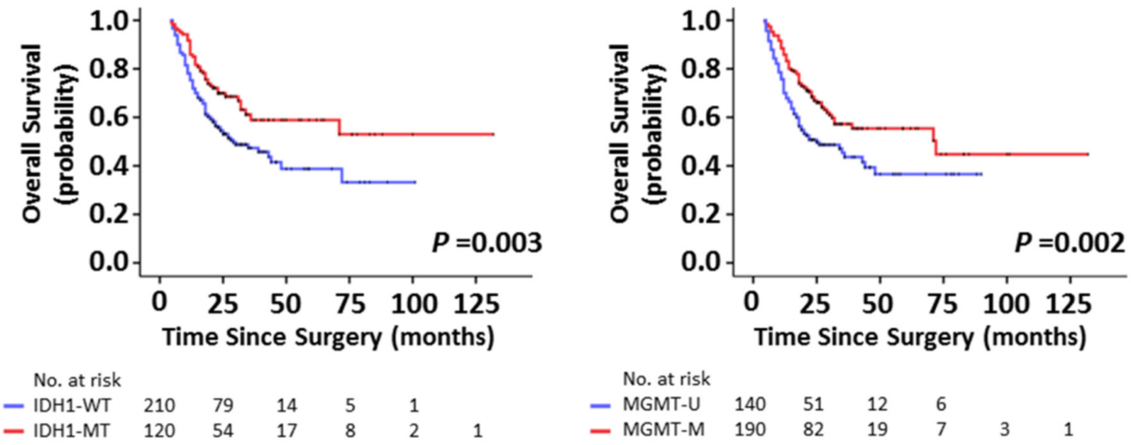

Supplementary Figure S6: *IDH1* and *MGMT* effect on survival in gliomas. Kaplan-Meier estimates of overall survival for the glioma patients by mutational status of *IDH1* mutations (left) and methylation status of *MGMT* promoter (right). WT and MT represent the *TERT* promoter wild-type and –mutated tumors, respectively. M and U represent the presence of methylated and unmethylated genes, respectively.
